# Pressure-Pair-Based Floor Localization System Using Barometric Sensors on Smartphones

**DOI:** 10.3390/s19163622

**Published:** 2019-08-20

**Authors:** Chungheon Yi, Wonik Choi, Youngjun Jeon, Ling Liu

**Affiliations:** 1 Department of Information and Communication Engineering, Inha University, Incheon 22212, Korea; 2 Papaya Co., Ltd., Incheon 22106, Korea; 3 College of Computing, Georgia Institute of Technology, Atlanta, GA 30332, USA

**Keywords:** floor localization, barometer, barometric pressure, relative pressure map, iterative optimization technique

## Abstract

As smartphone technology advances and its market penetration increases, indoor positioning for smartphone users is becoming an increasingly important issue. Floor localization is especially critical to indoor positioning techniques. Numerous research efforts have been proposed for improving the floor localization accuracy using information from barometers, accelerometers, Bluetooth Low Energy (BLE), and Wi-Fi signals. Despite these existing efforts, no approach has been able to determine what floor smartphone users are on with near 100% accuracy. To address this problem, we present a novel pressure-pair based method called FloorPair, which offers near 100% accurate floor localization. The rationale of FloorPair is to construct a relative pressure map using highly accurate relative pressure values from smartphones with two novel features: first, we marginalized the uncertainty from sensor drifts and unreliable absolute pressure values of barometers by paring the pressure values of two floors, and second, we maintained high accuracy over time by applying an iterative optimization method, making our method sustainable. We evaluated the validity of the FloorPair approach by conducting extensive field experiments in various types of buildings to show that FloorPair is an accurate and sustainable floor localization method.

## 1. Introduction

Ever since smartphones were equipped with barometers, starting in 2012, numerous research efforts on floor localization have tried to improve the accuracy of identifying a smartphone user’s floor location in a multi-floor building. Most of those efforts have succeeded only in detecting floor changes or the number of floors changed instead of determining the exact floor number. This is due to barometer limitations, such as sensor drifts, temporal variations, and unreliable pressure readings. With recent advances in micro-electro-mechanical systems (MEMS) technology, however, modern MEMS barometers have low power consumption, low cost, and very high-performance sensors. Because of these beneficial characteristics, barometers are now found in an abundance of mobile devices, including smartphones and tablets.

Despite all these opportunities, the floor localization problem remains a big challenge, since no existing methods identify a floor number with near 100% accuracy—a critical requirement for various applications, such as emergency location service, worker location tracking service, and clinical monitoring applications.

To cope with this problem, our goal was to design and implement a novel floor localization method that is able to estimate the exact floor number on which a smartphone user is located. Concretely, we argue that it is important to rethink the floor localization framework by exploring a relative pressure map [1] to maximize accuracy. The relative pressure map is a one-dimensional array containing pressure differences between the reference floor and the other floors. Note that the reference floor may be the same as or different from the first (ground or entry) floor. If a building has one entry point on the first floor, the first floor will be the reference floor in most cases for small buildings. However, because most buildings have many entry points on multiple floors, the reference floor may not be the first floor or one of the entry floors. In this paper, we define the reference floor as a logical floor that plays the role of a reference point in a relative pressure map.

Once we build this relative pressure map, we are able to compute the exact floor number using the difference between the pressure of the reference floor and the current floor as an index into the relative pressure map. With these observations in mind, we present a pressure-pair-based floor localization method called FloorPair, which constructs a relative pressure map for a building and thus offers near 100% accuracy under various environmental conditions.

Our contributions in this paper are:

(1) We propose a novel pressure-pair-based approach called FloorPair for constructing a relative pressure map. FloorPair makes use of pressure pairs between the reference floor and other floors in a multi-floor building and thus aggregates those pairs into a list of pressure differences, i.e., a relative pressure map.

(2) We introduce the marginalization of sensor drifts and absolute pressure errors when computing pressure differences. With this marginalization of unreliable characteristics, we construct a relative pressure map.

(3) We present an iterative optimization method based on the framework of the EM (expectation and maximization) algorithm to track pressure changes due to weather conditions in real-time. Using this method, we eliminate accumulated errors over time and provide a reliable and sustainable floor localization service.

(4) Through extensive experiments, we show that FloorPair offers a near 100% accurate floor localization result, and is an alternative for critical applications, such as emergency location service, worker location tracking service, and clinical monitoring applications.

The rest of the paper is organized as follows: The next section gives an overview of the related work in floor localization using the barometer and our motivations. The following Section 3 presents the advancements in modern barometers and new application opportunities. Section 4 touches on the efficacy of the barometer for floor localization and describes our marginalization of sensor drifts and unreliable absolute pressure readings. In addition, this section describes how the design of our FloorPair method efficiently constructs a relative pressure map and how it maximizes floor localization accuracy. We present the performance results of FloorPair in Section 5 and finally present our conclusion and future work in Section 6.

## 2. Related Work

### 2.1. Barometers in the Floor Localization Problem

After barometers started appearing in smartphones and tablets in 2012, Muralidharan et al. [1] analyzed their characteristics in smartphones, such as the Samsung Galaxy S4, Google Nexus 4, and Google Nexus 10. They observed that absolute pressure readings are unreliable indicators for floor localization, while the pressure differences between two floors are relatively consistent and steady measurements independent of time and location. Using these features, they proposed a method that determines whether the user has changed floors and that also estimates the number of floors changed. However, they concluded that it is hard to determine the actual floor number on which a user is located using the barometer. Banerjee et al. [2] proposed an unsupervised probabilistic learning method for floor localization, which combines the floor transition information with the WiFi-based localization method called Horus [3] to infer the current floor of a user and improve overall localization accuracy. Their approach identifies only the number of floors changed, and depends on the accuracy of the Horus system to detect the floor number, which suffers from problems inherited from learning techniques.

Ye et al. [4] proposed a crowdsourcing-based floor localization method using barometers. This method built a barometer fingerprint map using crowdsourcing and did not require Wi-Fi infrastructure and wardriving of the entire building. However, this method requires as many encounters in an elevator as possible, which limits the effectiveness of the crowdsourcing. Moreover, this method’s barometer reading clustering is based on timestamps, making this method impractical because, in reality, we cannot collect all barometer readings with all timestamps using crowdsourcing.

Some initial studies such as Skyloc [5], RADAR [6], and Place Lab [7] proposed a user location and tracking system using only radio frequency (RF) signals, while other studies [8,9,10] have started to fuse radio signals with barometers. None of these methods are applied to the floor localization problem because not all buildings have sufficient RF signals.

Ichikari et al. [11] proposed a method for estimating the floor level by decomposing the observed pressure into three components, i.e., device-specific offsets, environmental trends, and the altitude-dependent component. This method is similar to our method in that it is based on relative changes of atmospheric pressure values, but differs in that it utilizes beacons or Wi-Fi access points. Moreover, the accuracy of this method is dependent on the number of participants. Xu et al. [12] proposed a floor localization method that fuses inertial and barometric pressure measurements. However, this method required a special device mounted on the waist of a user, making this method impractical.

### 2.2. Detecting Users’ Entrance into a Building

The detection of a user entering a building is critical to the floor localization problem. If we know the moment when a user enters a building, it means that we are able to acquire the current pressure on that floor and use it to construct a relative pressure map. Naïve approaches to detect users at a gate would be to use pre-installed sensors, such as Radio Frequency Identification (RFID), Bluetooth Low Energy (BLE) beacons, or Near-Field Communication (NFC). In [11], they obtained specific floor-level information from localization infrastructures, such as beacons and Wi-Fi access points (APs). Yi et al. [13] proposed a visualized signal image-based method for detecting users’ entrance of a building. They used all signals that are received indoors and outdoors from smartphones by visualizing those signals in one signal image. Their proposed method constructs constellation images for specific indoor and outdoor locations and detects whether users are indoors or outdoors by learning the images with deep learning techniques. In this paper, we use this method for detecting a user’s entrance at a floor and triggering the current pressure measurement on that floor.

### 2.3. Motivations

As described in the previous section, no previous approach has been able to detect the exact floor number on which users are located accurately enough to support critical applications. There are four main reasons for this:

Firstly, the previous approaches did not determine the reference pressure because there was no method to detect users’ entrance without pre-installed sensors. Second, let us assume that the barometric pressure at a gate in a building is the reference pressure, *P*_ref_. If we know *P*_ref_, we can easily calculate the altitude *h* of a smartphone user as *h* = (*P*_ref_ − *P*_cur_)/0.12, where *P*_cur_ is the barometer reading on the current floor and the value of 0.12 hPa is the pressure decrease when going up every 1 m in a vertical direction. If each floor has the same height of h_0_, we then know that the floor number is *h*/ *h*_0_. The problem here was that *h*_0_ varies for different buildings. Third, the barometer reading *P*_cur_ from a smartphone was not accurate because of sensor drift. The sensor drift for the same floor level and even for the same model of smartphone reached 2 hPa, which led to a floor localization error ranging up to five stories. Fourth, they did not handle temporal pressure variations due to weather conditions and time.

To address these problems, we propose a pressure-pair-based approach called FloorPair for constructing a relative pressure map. The FloorPair method collects a minimum number of pressure pairs between the reference floor and some specific floors and aggregates those pairs into a list of pressure differences, i.e., a relative pressure map. While constructing the relative pressure map, we marginalize the sensor drift and unreliable absolute pressure errors. Once we have the relative pressure map for a building, our iterative optimization method is run to track pressure changes over time due to weather conditions in real-time and thus makes our method reliable and sustainable.

## 3. New Characteristics of Modern Barometers on Smartphones

### 3.1. High and Consistent Pressure Sensing Accuracy

**Figure 1:**
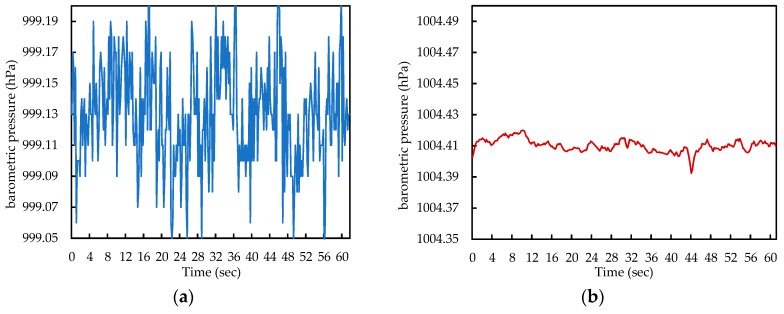
Noise differences between 2014 and 2018 smartphones. (**a**) Samsung Galaxy Note 4; (**b**) LG V40 ThinQ.

To clearly show the improvement in pressure sensing accuracy of modern barometers, we present the noises from Samsung Galaxy Note 4, released in 2014 and LG V40, released in 2018. As shown in Figure 1, the barometer on the more recent smartphone produces low noise and thus has a lower standard deviation in pressure measurements, as shown in Table 1.

### 3.2. Constant Pressure Difference between Two Floors

In addition to their low noise characteristics, recent barometers have become more accurate in relative pressure values. Although recent barometers still suffer from sensor drift and unreliable absolute pressure measurements, the difference of the barometer readings between any two floors has become more constant.

To demonstrate this characteristic, we conducted experiments in a university building, the Hi-Tech Center at Inha University, with four smartphones under high and low pressure conditions. Specifically, the smartphones used in the experiment were the LG V40, V10, and two Samsung Note 5s. We used two Note 5s to show that there are non-negligible errors in pressure readings, even from two devices of the same company and model. The building has 15 stories whose heights are 5 m for the basement floor, 4.5 m for the ground floor, and 3.9 m from the second floor to the 15th floor.

**Figure 2:**
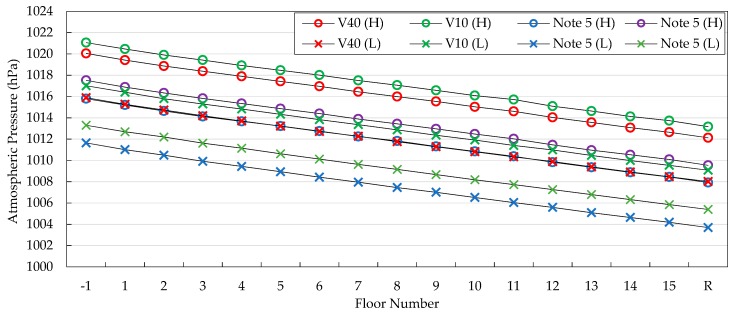
Steady and constant pressure differences across various devices and weather conditions.

As shown in Table 2 and Figure 2, the pressure differences are steady and in-sync across different devices and different weather conditions. In order to clearly show this characteristic, we present the differences between two floors and their standard deviations in Table 3.

### 3.3. Challenges for Constructing a Relative Pressure Map

**Figure 3:**
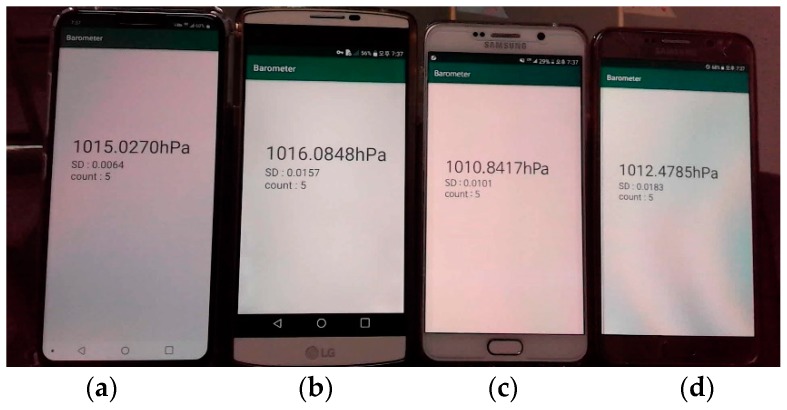
Pressure differences at the same place: (**a**) LG V40; (**b**) LG V10; (**c**) Samsung Note 5; (**d**) Samsung Note 5.

Even though modern barometers show the advancements described in the previous subsections, the barometers on recent smartphones still have the problem of sensor drift, which is a key challenge in constructing a relative pressure map. To clarify this characteristic, we took pressure readings at the same place and time using the LG V40, V10, and two Samsung Note 5s, as shown in Figure 3. We can see that the four devices display four different pressure values and also that the maximum difference is 1.4 hPa, which may result in a floor localization error ranging up to four stories in a typical building.

Through Figure 3, we can observe that the barometers still have an inherent drift from the real atmospheric pressure and that the drift varies even between two devices of the same company and model. In addition, Figure 3 shows that the absolute pressure measured by modern barometers is still unreliable. This unreliable characteristic of absolute pressure is also seen in Figure 2. Hence, to make use of the barometer pressure readings, it is important to calibrate sensor drift and absolute pressure readings. Based on these observations, we develop a novel algorithm to marginalize these two uncertainties and thus construct a relative pressure map, as will be described in Section 4.

## 4. Design and Implementation of FloorPair

In this section, we describe a pressure pair based floor localization called FloorPair, which aims to determine the exact floor number on which smartphone users are located. We designed FloorPair to achieve three goals: first, we constructed a relative pressure map with minimum costs; second, using the relative pressure map, we provided near 100% accuracy in determining the exact number of the floor; third, we maintained 100% accuracy over time for the sustainability of the floor localization service.

We first give a description of the variables used in this paper in Table 4.

A pressure value at each floor is estimated by Equation (1):

*P*(*f*) = *P*_ground_ + *drift* + *FP*(1, *f*). (1)

Let us assume that *P*_ground_ is the ground truth pressure of the reference floor and that we want to know a floor pair, *FP*(1, *f*). Note that, as described in Section 1, the reference floor may or may not be the first floor. For simplicity, however, we assume that the reference floor corresponds to the first floor in Equation (1). Then, as described in Table 4, *FP*(1, *f*) denotes the pressure difference between the reference floor (1) and the *f*-th floor. We construct a relative pressure map using a set of these *FP*s.

However, it is almost impossible to obtain the exact value of the sensor drift because every smartphone has a different value of drift, as described in Section 3.3. In addition, it is also impractical to measure *P*_ground_ because we need a pre-installed and high-cost barometer on the ground floor. Therefore, in order to obtain a floor pair, we need to marginalize these two variables. For example, if we want to know *FP*(1, *f*), we get it by subtracting Equation (2) from Equation (3):

*P*(1) = *P*_ground_ + *drift* + *FP*(1,1), (2)

*P*(*f*) = *P*_ground_ + *drift* + *FP*(1, *f*). (3)

Then, *FP*(1, *f*) can be obtained by the following equation, since *FP*(1,1) is zero:

*FP*(1, *f*) = *P*(*f*) − *P*(1). (4)

Our goal was to construct a relative pressure map using these *FP*s. Given a set of *FP*s, we may think that we easily construct a relative pressure map. Specifically, once we collect all pressure values at every floor, *P*(*f*), we simply build a relative pressure map by subtracting *P*(1) from *P*(*f*).

However, this naïve approach does not intuitively work for three reasons. First, pressure value varies, even during the process of pressure collection on each floor. This means that only pressure values collected within a time threshold *T* are valid when obtaining floor pairs. Second, we need to reduce the cost of collecting pressure values for practical reasons. We aimed at constructing a relative pressure map with minimal cost. Third, even after successfully constructing a relative pressure map, we had to iteratively update the value of *P*_ref_ because pressure values such as *P*_ref_ and *P*_cur_ continued to vary over time. In the next three subsections, we address each of these three issues.

### 4.1. Pressure Variations in Minutes

Barometric pressure varies often enough to change every minute. To show this characteristic, we use the dataset of the pressure measurements in Seoul in February 2019 [14]. Table 5 summarizes the number of pressure changes in *n* minutes. For example, there were 20 cases where pressure variation in 1 minute was greater than 0.2 hPa. Similarly, the number of pressure variations greater than 0.3 hPa in 3 minutes was 94.

Table 5 shows that a pressure value measured at a specific time *t* and a pressure value measured at *t* + *T* cannot be paired for calculating a relative pressure value. In other words, a floor pair is established only among the pressure values measured at times within a time threshold *T*. For small and low buildings, we may collect all pressure values required to construct a relative pressure map within *T*. In this case, we simply construct relative pressure maps for such buildings by calculating the differences between *P*_ref_ and *P*(*f*). However, in reality, we have to take into account the case where we cannot collect the required pressure values within *T*. To address this problem, we developed the FloorPair algorithm.

### 4.2. FloorPair: Generating a Relative Pressure Map from Collected Pressure Data

An input of the FloorPair algorithm is a set of collected pressure tuples consisting of floor number, pressure reading, and its timestamp (line 1 in Algorithm 1). Using these tuples, we generated a valid set of floor pairs using only tuples collected within *T*. Specifically, we generated a floor pair *FP*(*f*_pivot_, *f*_probe_) with a condition where *f*_pivot_ was greater than *f*_probe_ and the difference of their timestamp was less than *T* (line 6 in Algorithm 1).
**Algorithm 1. Generating a set of pressure pairs**1 2 3 4 5 6 7 8 9 10 11input: *collected_pressure_set* //A set of tuples, containing (floor, pressure readings, timestamp) output: *FP_set* // A set of FloorPairs, *FP*(*f*_pivot_, *f*_probe_) for a tuple *Q* ∊ *collected_pressure_set* // self-join for a tuple R ∊ collected_pressure_set  if *Q*.floor < *R*.floor and |*Q*.timestamp − *R*.timestamp| < *T* (= 5 min)    *FP*(*Q*.floor, *R*.floor) ← *R*.pressure – *Q*.pressure     Add *FP*(*Q*.floor, *R*.floor) to *FP_set* Sort *FP_set* on *f*_pivot_, *f*_probe_ in { 1, −1, 2, −2, 3, −3 … } order // Floor order closer to the reference floor return *FP_set* // return to Algorithm 2

To illustrate the algorithm for constructing a set of floor pairs, we present a running example in Table 6 and Table 7. Note that we used a special order that represents a floor order to consider floors close to the reference floor first, i.e., {1, −1, 2, −2, 3, −3..}. To implement this floor order, we added 0.5 to the absolute value of negative floor numbers. For example, a basement floor B1 is greater than 1F and is less than 2F because the floor number of B1 is converted to 1.5.

When Q = (1, 1000, 12:01), the possible tuples to be paired are (−1, 1001, 12:01), (2, 999, 12:04), (6, 995, 12:02) collected before 12:01 + *T*(= 5 min), satisfying the condition in line 6. Note that we heuristically set *T* to 5 minutes based on Table 5. These three tuples resulted in three floor pairs, *FP*(1, −1), *FP*(1,2), and *FP*(1,6), as shown in the first three rows in Table 7. When Q = (3, 991, 13:01), the possible tuple is (4, 990, 13:00), resulting in *FP*(3,4).

After constructing a set of floor pairs, *FP_set*, we sorted it in the floor order for Algorithm 2, as shown in line 10 in Algorithm 1.
**Algorithm 2. Constructing a Relative Pressure Map**1 2 3 4 5 6 7 8 9 10 11 12 13 14 15 16 17 18 19 20 21input: *FP_set* output: *Diff_map* Initialize *Diff_map*[*f*_top_ − *f*_bottom_], *pivot_floors*[*f*_top_ − *f*_bottom_] to 0 Set *Diff_map*[1] ← 0, *pivot_floors*[1] ← 1 for a tuple *R* ∊ *FP_set* // Forward merging from pivot floors if *pivot_floors*[*R*.*f*_pivot_] ≠ 0 and *pivot_floors*[*R*.*f*_prove_] = (0 or *R*.*f*_pivot_)   Update *Diff_map*[*R*.*f*_probe_] after including (*Diff_map*[*R*.*f*_pivot_] + *FP*(*f*_pivot_, *f*_probe_))   *pivot_floors*[*R*.*f*_prove_] ← *R*.*f*_pivot_; for a tuple *R* ∊ *FP_set* // Backward merging from probe floors if *pivot_floors*[*R*.*f*_prove_] ≠ 0 and *pivot_floors*[*R*.*f*_pivot_] = (0 or *R*.*f*_prove_)   Update *Diff_map*[*R*.*f*_pivot_] after including (*Diff_map*[*R*.*f*_prove_] − *FP*(*f*_pivot_, *f*_probe_))   *pivot_floors*[*R*.*f*_pivot_] ← *R*.*f*_prove_; for *f* in range − *f*_bottom_ ~ *f*_top_ // Complete a relative pressure map using linear interpolation if *pivot_floors*[*f*] = 0 // Fill a zero item with a linear interpolated value   *Diff_map*[*f*] ← linear interpolation between two nearest non-zero values in *Diff_map* return *Diff_map* // return to Algorithm 3

Algorithm 2 constructs a relative pressure map, *Diff_map*, using *FP_set* from Algorithm 1. First, we initialized *Diff_map*, *pivot_floors*, as shown in line 1~5 in Algorithm 2. Each column named “initial state” in Table 8 and Table 9 shows the initial values of *Diff_map* and *pivot_floors*, respectively. Each element of the *pivot_floors* array contains its corresponding pivot floor number. Note that the bold and underlined numbers denote the values updated at that stage.

At the stage of “Merging (1,x)”, there were three pairs whose pivot floor was 1, i.e., (1, −1), (1,2), and (1,4) in *FP_set*. Then, the values for the −1, 2, 4 floors in *Diff_map* were updated to 1, −1, and −5, respectively (line 9 in Algorithm 2). In addition, in line 10 in Algorithm 2, the pivot floor numbers in the *pivot_floors* array were updated to 1. At the next stage of “Merging (−1,x)”, there were two pairs whose pivot floors were −1, (−1,2), and (−1,6). However, because the value of the probe floor 2 already had the relative pressure −1, the *FP*(−1,2) did not change the value of the probe floor 2. Similarly, the *FP*(−1,6) did not update the value of the probe floor 6, −5. Therefore, there were no changes at “Merging (−1,x)”.

**Figure 4:**
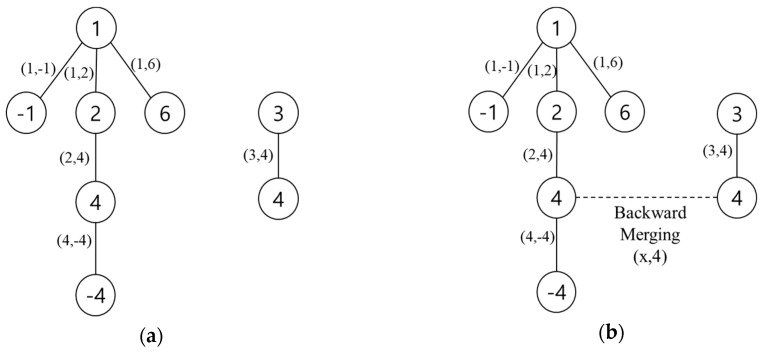
Pair relationship tree after forward and backward merging: (**a**) tree after forward merging; (**b**) tree after backward merging.

At the “Merging (3,x)” stage, there was one *FP*(3,4). However, it cannot change any values because the pivot value of 3 in the *pivot_floors* array was 0, which means that there was no connection to the reference floor. Figure 4a illustrates the pair relationship trees after the first for-loop in line 7~10 in Algorithm 2. As shown in Figure 4a, it was possible that the pair relationship trees were disconnected and thus formed a forest. In other words, the *FP*(3,4) had no connection to the reference floor, 1, which meant that there was no relation to calculate the pressure difference between the reference floor and floor 3. To prevent this case, we examined a pair relationship again but in reverse by swapping the role of the probe floor and the pivot floor in line 12~15 in Algorithm 2. In this backward merging step, we connected all separate trees by searching for a relationship between the probe floor (instead of the pivot floor) and the reference floor. The dashed line in Figure 4b shows such a relationship between floor 4 and the reference floor.

After two merging steps, we filled zero values with linear interpolated values between two nearest non-zero values in *Diff_map* (line 17~19 in Algorithm 2). Then, we completed a relative pressure map for a building, as shown in the column labeled “Final” in Table 8.

### 4.3. Estimating the Exact Floor Number and Refreshing the Reference Pressure

Once we obtained a relative pressure map, *Diff_map*, we estimated the number of the current floor on which a user is located simply by subtracting *P*_ref_ from *P*_cur_ (line 11~12 in Algorithm 3), where *P*_cur_ is a pressure reading from a user’s smartphone and *P*_ref_ is the reference pressure obtained in line 5 in Algorithm 3. As mentioned in Section 2.2, we detected the entry floor, *f*_entry_, using [13] and then calculated *P*_ref_ as shown in line 5 in Algorithm 3.
**Algorithm 3. Estimating the Current Floor Using *Diff_Map***1 2 3 4 5 6 7 8 9 10 11 12 13 14 15 16 17 18input: *Diff_map* output: *f*_cur_  *P*_cur_ ← current barometer reading  // Initialize *P*_ref_ using *f*_entry_ from [13]  *P*_ref_ ← *P*_cur_ – *Diff_map*[*f*_entry_]   While(TRUE)   *P*_cur_ ← current barometer reading    // E(expectation)-step: Estimate *f*_cur_ based on *P*_ref_   *P*_diff_ ← *P*_cur_ - *P*_ref_   *f*_cur_ ← index of the value closest to *P*_diff_ in *Diff_map*   Report *f*_cur_ to the caller    // M(maximization)-step: Update *P*_ref_ based on *f*_cur_
   if IsUpdate() ==TRUE    // if a user does not move vertically,     *P*_ref_ ← *P*_cur_ − *Diff_map*[*f*_cur_] // then update the reference pressure *P*_ref_ in accordance with             // the current pressure, *P*_cur_ on *f*_cur_.

However, the issue that makes the floor localization problem difficult is that *P*_cur_ continues to vary over time. For example, let us assume that a user is on a floor at a specific time *t* and its *P*_cur_ is 1000 hPa. After the user stays on the same floor for a couple of hours after *t*, *P*_cur_ may have changed to 1020 hPa. This means that the value of (*P*_cur_ − *P*_ref_) is no longer valid to estimate the exact floor number from *Diff_map* and thus we need to iteratively update the value of *P*_ref_ in accordance with the variations of *P*_cur_, as shown in line 16~17 in Algorithm 3. Specifically, if a user moves only horizontally and not vertically for a certain time period *s*, the value of *P*_ref_ is updated in accordance with the relative pressure value on *f*_cur_ in *Diff_map* (line 16~17 in Algorithm 3).

Algorithm 4 shows the IsUpdate() function. In order to determine whether a user moves vertically or not, we take into account two parameters: the pressure variations, *p*, allowed for a time period *s*.
**Algorithm 4. IsUpdate() – Determining Whether We Need to Update *P*_ref_ or Not**1 2 3 4 5 6 7 8 9 10 11 12 13 14input: *P*_cur,_
*f*_cur,_
*Diff_map* output: TURE, FALSE  *p* ← (*Diff_map*[*f*_cur_] − *Diff_map*[*f*_cur_ + 1])/2 *s* ← *p*/0.015 // Based on the lower and upper bound if Δ*P*_cur_ > *p* in *s* seconds // if a user’s vertical movement is out of the *true range*,           // then return FALSE. return FALSE // FALSE means “Don’t update *P*_ref_ because a user is moving vertically“. if |*Diff_map*(*f*_cur_) − (*P*_cur_ − *P*_ref_)| > *p/2* // if a user stays out of the *true range*, then return FALSE. return FALSE return TRUE // Otherwise, “Update *P*_ref_ based on *f*_cur_“.

Since people usually live on the surface of a floor, we consider the range of vertical displacement of a user’s smartphone as half of the typical floor height. On the basis of this fact, we set *p* to the half of a pressure difference between two floors, (*Diff_map*[*f*_cur_] − *Diff_map*[*f*_cur_ + 1])/2 (line 4 in Algorithm 4).

**Figure 5:**
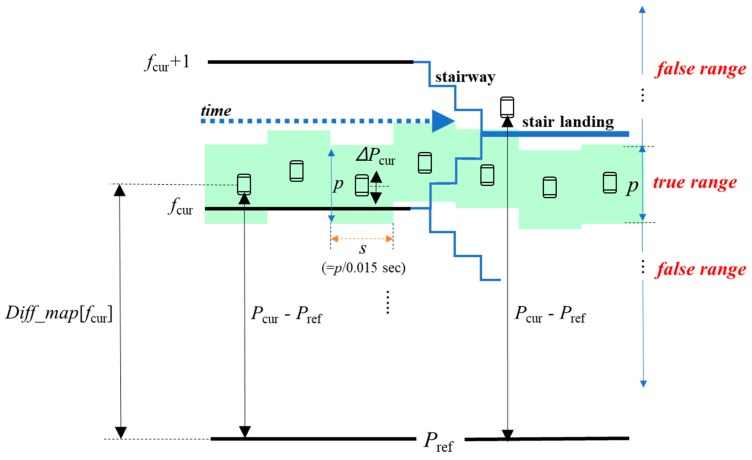
Illustration of Algorithm 4.

To determine whether a user is moving vertically or not*,* we divided a floor height into two ranges, i.e., *true range* and *false range*, as shown in Figure 5. The goal of the IsUpdate() function is to return TRUE only if it decides that a user is vertically stable within *p* (i.e., in the *true range*) for *s* seconds. Then, our algorithm updates *P*_ref_ (line 17 in Algorithm 3). Otherwise, the IsUpdate() function returns FALSE, which means that our algorithm needs not to update *P*_ref_ because a user is still moving vertically by taking an elevator/escalator or using the stairs. The IsUpdate() function also returns FALSE if a user is in the *false range*, even though the user stops for *s* seconds in the middle of stairs, e.g., a stair landing. This is to avoid wrong updates of *P*_ref_ while a user is moving vertically very slowly. To address this issue, we introduced a lower bound of the parameter *s*.

The lower bound was based on the slowest vertical movement speed of a human, 0.2 m/s [15]. Therefore, the lower bound is calculated by 0.2 (m/s)/8 (m/hPa) = 0.025 hPa/s.

The upper bound of *s* is derived from Table 5. If *s* is too long, it means that we cannot distinguish weather changes from users’ vertical movements. Therefore, the upper bound corresponds to the maximum time period over which no change in atmospheric pressure occurs. As shown in Table 5, the number of pressure variations greater than 0.3 hPa is zero within 1 minute, which means that the upper bound is calculated by (0.3 hPa / 60 sec) = 0.005 hPa/s.

The parameter *s* is determined by *p* / ((lower bound + upper bound)/2), i.e., *p*/0.015. For example, if *p* is the half of a typical floor pressure difference [1,12], 0.2 hPa, the parameter of *s* is 13 seconds. Since the parameter *s* is a function of *p*, the parameter *s* reflects the height of the floor.

In the case where *s* exceeds the upper bound, there is a possibility according to Table 5 that a pressure change coming from weather changes is mistaken for a pressure change caused by an actual user’s vertical movement. On the other hand, if *s* is below the lower bound and a user moves vertically very slowly (e.g., below 0.2 m/s), our method continues to make unnecessary updates of *P*_ref_. On the basis of these lower and upper bound characteristics, we set *s* to *p*/0.015 seconds in order to provide 100% accuracy, as shown in Section 5.

As shown in Algorithms 3 and 4, our algorithm is based on an iterative optimization method such as the expectation–maximization (EM) algorithm [16], which is widely used in statistics and data clustering in machine learning, to jointly optimize two parameters. Conceptually, when it is hard to optimize two interacting parameters A and B at the same, the EM algorithm optimizes parameter A while holding parameter B’s value. In the next step, the EM algorithm optimizes parameter B based on parameter A from the previous step. The EM algorithm basically alternates between performing an expectation (E) step and a maximization (M) step. 

**Figure 6:**
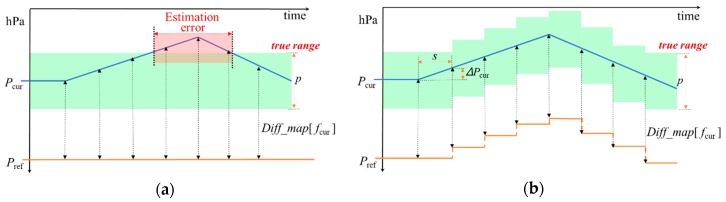
Graphic representation of our iterative optimization method: (**a**) before applying our optimization method; (**b**) after applying our optimization method.

In the E-step of our algorithm, we estimated an initial *f*_cur_ using *P*_ref_. Then, in the M-step, conversely, we optimized *P*_ref_ using the *f*_cur_ estimated in the E-step. This new *P*_ref_ is iteratively used to estimate *f*_cur_ in the next E step. We present an illustration of our optimization method in Figure 6.

Because our algorithm is based on the concept of the EM algorithm, we can easily apply recent machine learning techniques to the floor localization problem and we will leave this as future work.

## 5. Performance Evaluations

### 5.1. Collecting Pressure Data

One of our goals was to minimize the cost of collecting pressure data. It is important for the floor localization service to be widely deployed in practice with minimum cost for both construction and maintenance. Our FloorPair method basically needs to collect floor pairs to construct a relative pressure map for a building. Floor pairs are easily collected by reading the numbers of floors in a building and the pressure values and time of a smartphone without special devices or experts.

As described in the following two paragraphs, this collection process is performed only once by anyone who wants to provide the exact number of floors of a building, e.g., the owner of a building, a service provider, or an end-user.

If the floors of a building have the same height, we need only one floor pair. For example, in the case of a university building whose floors have the same height, we constructed a relative pressure map by collecting one pressure value at the first floor and one at the top floor, resulting in *FP*(1,15), as shown in Table 10. With this one *FP*, we can build a relative pressure map for this building and thus obtain the exact floor number with 100% accuracy.

On the other hand, a tall commercial building such as the POSCO tower (B1 ~ 65F) has different heights for different floors. In this case, we need a couple of floor pairs to complete a relative pressure map, as shown in Table 10. First, we collected two pressure values on the first and top floor. Then, we calculated a floor localization error as shown in the column of “1st Error” of Table 10. To correct this error, we collected the 2nd pair, *FP*(1,2), the 3rd pair, *FP*(1,14), and the 4th pair, *FP*(1,36). With these four floor pairs, we easily constructed a relative pressure map for a 66-story building with minimum cost. 

Note that once the relative pressure map of a building is completed, it means that it is unique and permanent for that building, and does not need to be collected repeatedly.

### 5.2. Result of Experiments

As shown in Table 10, our method achieves 100% accuracy by iteratively correcting the errors. Once the relative pressure map is completed, our method also maintains 100% accuracy by optimizing the reference pressure in accordance with the current pressure using the framework of the EM (expectation and maximization) algorithm as described in Section 4.3.

To further validate the efficacy of our method, we conducted extensive field experiments in the various buildings, as listed in Table 11.

Among the five smartphone models in Table 12, we used one or two models to collect floor pairs and five models to evaluate the accuracy of our proposed method, as shown in Table 13. We used different models of smartphones for both collection and evaluation to show that our method works well even when different smartphones are used for collection and evaluation. As shown in Table 14, our method shows 100% accuracy, independent of types of phones and buildings. For each trial, we counted success only when our method consistently showed the exact number of a floor for about 10 minutes to reflect constantly changing pressure values, such as *P*_cur_ and *P*_ref_. Note that *P*_cur_ is measured about 600 times in 10 minutes, while *P*_ref_ is updated 20 to 50 times depending on the height of a floor.

Moreover, in order to further evaluate the sustainability of our method, we conducted the same experiments up to five times for several months. As shown in Table 14, our method maintains 100% accuracy over time. In this paper we used the mean absolute error (MAE) metric defined by MAE $=\frac{1}{n}\sum_{1}^{n}\left|\left(estimated\;floor\;number\right)-\left(actual\;floor\;number\right)\right|$, where *n* is the number of tests, to evaluate the accuracy of our method.

### 5.3. Reasons for near 100% Accuracy

Even though our FloorPair method showed 100% accuracy in our experiments, our method is said to provide *near* 100% accuracy for the following reasons: First, we did not conduct our experiments under serious weather conditions, such as tornadoes and hurricanes. In this case, we do not expect that our method provides 100% accuracy. Second, if a user continues to move up and down for a long time, e.g., 5 minutes, it causes about 0.017% error according to Table 5. Third, if a user vertically moves below 12 cm per second, our method mistakes a user’s vertical movement for a weather change, leading to erroneous results. In the second and third cases, our method cannot provide 100% accuracy only to users moving with these extreme patterns, but it continues to provide near 100% accuracy to other users.

## 6. Discussion

In this paper, we proposed a pressure-pair-based floor localization method called FloorPair that aims at determining the exact number of the floor on which smartphone users are located. Specifically, we had the following three goals for the floor localization problem: first, we construct a relative pressure map with minimum costs; second, using the relative pressure map, we provide near 100% accuracy in determining the exact number of a user’s floor; third, we maintain near 100% accuracy over time for sustainability of the floor localization service.

To achieve these goals, FloorPair first generates a set of pressure pairs from a dataset of pressures collected in a building. In the process of this collection, FloorPair needs only a few pressure readings on a minimum number of floors, unlike previous approaches. Using this set of pressure pairs, FloorPair merges those pressure pairs into a relative pressure map that contains pressure differences between a reference floor and the other floors in the building. On the basis of this relative pressure map, FloorPair determines the exact floor number on which users are located with near 100% accuracy. In addition, FloorPair is able to maintain this high accuracy over time with an iterative optimization method based on the framework of the EM algorithm, making our method sustainable.

Extensive field experiments in various types of buildings show that FloorPair is near 100% accurate and is a sustainable floor localization method with minimum costs. For future work, we plan to augment our method with recent artificial intelligence techniques to further expand the adaptability of our method to various environments.

## Figures and Tables

**Table 1:** Standard Deviations of 2014 and 2018 smartphones

Model	Standard Deviation
Samsung Galaxy Note 4 (2014)	0.034021
LG V40 ThinQ (2018)	0.003927

**Table 2:** Barometric pressures measured by various devices and weather conditions.

Floor	V40 (H)	V10 (H)	Note 5 (H)	Note 5 (H)	V40 (L)	V10 (L)	Note 5 (L)	Note 5 (L)
**−1**	1020.0485	1021.0597	1015.7985	1017.5154	1015.8888	1017.011	1011.6438	1013.3
**1**	1019.4166	1020.4616	1015.2037	1016.8817	1015.2662	1016.407	1011.0079	1012.694
**2**	1018.8644	1019.9028	1014.6411	1016.3334	1014.7261	1015.795	1010.4865	1012.2
**3**	1018.3719	1019.4202	1014.1196	1015.822	1014.1901	1015.297	1009.9165	1011.623
**4**	1017.8944	1018.9136	1013.6665	1015.3442	1013.6993	1014.826	1009.4372	1011.148
**5**	1017.4191	1018.4623	1013.2254	1014.8593	1013.213	1014.328	1008.9368	1010.62
**6**	1016.9572	1018.0136	1012.7502	1014.3963	1012.7152	1013.825	1008.4254	1010.121
**7**	1016.4421	1017.5021	1012.2452	1013.8857	1012.2665	1013.361	1007.9504	1009.623
**8**	1015.9894	1017.0611	1011.8245	1013.4329	1011.7593	1012.865	1007.45	1009.156
**9**	1015.5374	1016.5809	1011.3105	1012.9605	1011.2819	1012.364	1007.007	1008.666
**10**	1015.027	1016.0848	1010.8417	1012.4785	1010.8111	1011.913	1006.5177	1008.181
**11**	1014.6046	1015.7147	1010.3815	1012.0249	1010.3336	1011.401	1006.0361	1007.725
**12**	1014.0341	1015.0899	1009.8289	1011.4583	1009.8873	1010.982	1005.5866	1007.255
**13**	1013.5652	1014.6333	1009.3502	1010.9577	1009.389	1010.467	1005.0876	1006.788
**14**	1013.0734	1014.1267	1008.876	1010.5246	1008.9093	1009.987	1004.6375	1006.314
**15**	1012.6477	1013.7304	1008.4425	1010.089	1008.4609	1009.537	1004.1875	1005.847
R*	1012.1241	1013.1687	1007.9241	1009.5352	1008.0138	1009.075	1003.6888	1005.388

(H) High barometric pressure, (L) Low barometric pressure. * Rooftop.

**Table 3:** Pressure differences and standard deviations.

	V40 (H)	V10 (H)	Note 5 (H)	Note 5 (H)	V40 (L)	V10 (L)	Note 5 (L)	Note 5 (L)	Std. Dev.
**B1–1**	−0.6319	−0.5981	−0.5948	−0.6337	−0.6226	−0.6042	−0.6359	−0.6054	0.0159
**1–2**	−0.5522	−0.5588	−0.5626	−0.5483	−0.5401	−0.612	−0.5214	−0.4944	0.0318
**2–3**	−0.4925	−0.4826	−0.5215	−0.5114	−0.536	−0.4985	−0.57	−0.5768	0.0327
**3–4**	−0.4775	−0.5066	−0.4531	−0.4778	−0.4908	−0.4702	−0.4793	−0.4749	0.0144
**4–5**	−0.4753	−0.4513	−0.4411	−0.4849	−0.4863	−0.4988	−0.5004	−0.5278	0.0260
**5–6**	−0.4619	−0.4487	−0.4752	−0.463	−0.4978	−0.5022	−0.5114	−0.4992	0.0216
**6–7**	−0.5151	−0.5115	−0.505	−0.5106	−0.4487	−0.4645	−0.475	−0.4985	0.0234
**7–8**	−0.4527	−0.441	−0.4207	−0.4528	−0.5072	−0.4956	−0.5004	−0.4667	0.0291
**8–9**	−0.452	−0.4802	−0.514	−0.4724	−0.4774	−0.5008	−0.443	−0.4898	0.0220
**9–10**	−0.5104	−0.4961	−0.4688	−0.482	−0.4708	−0.4517	−0.4893	−0.4856	0.0169
**10–11**	−0.4224	−0.3701	−0.4602	−0.4536	−0.4775	−0.512	−0.4816	−0.4556	0.0399
**11–12**	−0.5705	−0.6248	−0.5526	−0.5666	−0.4463	−0.4184	−0.4495	−0.4698	0.0703
**12–13**	−0.4689	−0.4566	−0.4787	−0.5006	−0.4983	−0.5153	−0.499	−0.4672	0.0192
**13–14**	−0.4918	−0.5066	−0.4742	−0.4331	−0.4797	−0.4805	−0.4501	−0.4744	0.0215
**14–15**	−0.4257	−0.3963	−0.4335	−0.4356	−0.4484	−0.45	−0.45	−0.4669	0.0198
**15–R**	−0.5236	−0.5617	−0.5184	−0.5538	−0.4471	−0.4619	−0.4987	−0.4584	0.0410
SD *	0.0385	0.0617	0.0366	0.0386	0.0253	0.0271	0.0321	0.0312	

* Note that the standard deviations are calculated only using the values from the 2nd floor to the R(rooftop) floor because the heights from the B1 floor to the 2nd floor are different from each height from the 2nd floor to the R floor.

**Table 4:** Definition of variables.

Variables	Description
*f*	Floor number (bottom floor ≤ *f* ≤ top floor)
*f*_pivot_	Floor number of pivot floor
*f*_probe_	Floor number of floor to be probed paired with *f*_pivot_
*FP*(*f*_pivot_, *f*_probe_)	FloorPair: Pressure difference between a pivot floor *f*_pivot_ and a probe floor *f*_probe_
*P*(*f*)	Pressure reading on the *f*-th floor
*Diff_map*[*f*_top_ − *f*_bottom_]	An array for relative pressure differences for a (*f*_top_ − *f*_bottom_)-story building
*T*	Threshold for the valid time interval
*P*_cur_	Pressure reading at a place on which a user is located
*P*_ref_	Pressure value at the reference floor

**Table 5:** The number of pressure variations in *n* minutes.

	1 min	2 min	3 min	4 min	5 min	10 min	15 min	20 min	30 min	60 min
>0.6 hPa	0	0	0	1	4	92	366	1010	3399	11,810
>0.5 hPa	0	0	0	1	7	110	401	1145	3711	12,452
>0.4 hPa	0	1	6	17	60	497	1566	3310	7519	18,529
>0.3 hPa	0	14	94	254	444	2101	5010	8195	14,052	25,626
>0.2 hPa	20	325	939	1751	2724	8717	14,261	25,108	25,108	33,989

Total 40,320 minutes in 28 days.

**Table 6:** Input of Algorithm 1, *collected_pressure_set*.

Floor	Pressure	Timestamp
6	995	12:02
5	-	-
4	997, 990	12:08, 13:00
3	991	13:01
2	999	12:04
1	1000	12:00
−1	1001	12:01
−2	-	-
−3	-	-
−4	1004	12:11

**Table 7:** Output of Algorithm 1, *FP_set*.

*f*_pivot_, *f*_probe_	*FP*(*f*_pivot_, *f*_probe_)
1, −1	1
1, 2	−1
1, 6	−5
−1, 2	−2
−1, 6	−6
2, 4	−2
2, 6	−4
3, 4	−1
4, −4	7

**Table 8:** Construction of a relative pressure map, *Diff_map.*

Floor	*Diff_Map*							
	Initial State	Merging (1,x)	Merging (−1,x)	Merging (2,x)	Merging (3,x)	Merging (4,x)	Backward Merging (x,4)	Final
6	**0**	**−5**	−5	−5	−5	−5	−5	−5
5	**0**	0	0	0	0	0	0	**−4**
4	**0**	0	0	**−3**	−3	−3	−3	−3
3	**0**	0	0	0	0	0	**−2**	−2
2	**0**	**−1**	−1	−1	−1	−1	−1	−1
1	**0**	0	0	0	0	0	0	0
−1	**0**	**1**	1	1	1	1	1	1
−2	**0**	0	0	0	0	0	0	**2**
−3	**0**	0	0	0	0	0	0	**3**
−4	**0**	0	0	0	0	**4**	4	4

**Table 9:** *pivot_floors* while constructing *Diff_map.*

Floor	*Pivot_Floors*						
	Initial State	Merging (1,x)	Merging (−1,x)	Merging (2,x)	Merging (3,x)	Merging (4,x)	Backward Merging (x,4)
6	**0**	**1**	1	1	1	1	1
5	**0**	0	0	0	0	0	0
4	**0**	0	0	**2**	2	2	2
3	**0**	0	0	0	0	0	**4**
2	**0**	**1**	1	1	1	1	1
1	**1**	1	1	1	1	1	1
−1	**0**	**1**	1	1	1	1	1
−2	**0**	0	0	0	0	0	0
−3	**0**	0	0	0	0	0	0
−4	**0**	0	0	0	0	**4**	4

**Table 10:** Process of correcting floor localization errors.

Floor	Hi-Tech Center		Floor	POSCO Tower-Songdo							
	1st Pair	Final Error		1st Pair	1st Error	2nd Pair	2nd Error	3rd Pair	3rd Error	4th Pair	Final Error
**−1**		0	**−1**		0		0		0		0
**1**	●	0	**1**	●	0	●	0	●	0	●	0
**2**		0	**2**		+1	●	0		0		0
**3**		0	**3**		+1		0		0		0
**4**		0	**4**		+1		0		0		0
**5**		0	**5**		+1		0		0		0
**6**		0	**6**		+1		0		0		0
**7**		0	**7**		+1		0		0		0
**8**		0	**8**		0		0		0		0
**9**		0	**9**		0		0		0		0
**10**		0	**10**		0		0		0		0
**11**		0	**11**		−1		0		0		0
**12**		0	**12**		−1		0		0		0
**13**		0	**13**		−1		0		0		0
**14**		0	**14**		−1		+1	●	0		0
**15**	●	0	**...**								
			**36**		+2		+2		+2	●	0
			**...**								
			**65**	●	0		0		0		0

**Table 11:** Buildings used for our field experiments.

Name	Floors	Purpose	Type	Location
Lotte World Tower	123, −6	Office	Skyscraper	Seoul, Korea
POSCO Tower-Songdo	68, −3	Office	Skyscraper	Incheon, Korea
COEX	4, −2	Commercial	Wide and Flat	Seoul, Korea
HYUNDAI Department Store COEX Branch	11, −4	Commercial	Wide and Tall	Seoul, Korea
Gangnam Station	−2	Public	Underground	Seoul, Korea
Indeogwon Station	−2	Public	Underground	Anyang, Korea
I-first Tower	14, −7	Commercial	Narrow and Tall	Anyang, Korea
Woojung Town	9, −1	Commercial	Narrow and Tall	Anyang, Korea
Star Tower	15, −2	Commercial	Narrow and Tall	Anyang, Korea
Inha Hi-Tech Center	15, −1	University	Narrow and Tall	Incheon, Korea
Wellcounty 4 Apartment	30, −2	Residential	Narrow and Tall	Incheon, Korea
Woomin Villa	4, −1	Residential	Narrow	Incheon, Korea

**Table 12:** Smartphones used for our field experiments.

Model	Notation	Manufacturer	Year of Release
Galaxy Note 9	Note 9	Samsung	2018
V40 ThinQ	V40	LG	2018
V10	V10	LG	2015
Galaxy Note 5	Note 5_gold_	Samsung	2015
Galaxy Note 5	Note 5_white_	Samsung	2015

**Table 13:** Environment of experiments.

Name of Building	Devices Used For		Visited Floors	# of Floor Pairs Collected
	Collection	Evaluation		
Lotte World Tower	V40	V40, Note 5_gold_, Note 5_white_	−1~2 31,123	3
POSCO Tower-Songdo	V40	V40, Note 5_gold_, Note 5_white_	−1~14 36, 65	4
COEX	V10, V40	V40, V10, Note 5_white_	−2~4	4
HYUNDAI Department Store COEX Branch	V10, Note 5_white_	V40, V10, Note 5_white_	−4~ 11	5
Gangnam Station	V10, Note 5_white_	V40, V10, Note 5_white_	−2~1	1
Indeogwon Station	V10, Note 5_gold_	V40, V10, Note 9, Note 5_gold_, Note 5_white_	−2~1	1
I-first Tower	V10, Note 5_gold_	V40, V10, Note 9, Note 5_gold_, Note 5_white_	−7~14	21
Woojung Town	V10, Note 5_gold_	V40, V10, Note 9, Note 5_gold_, Note 5_white_	−1~9	15
Star Tower	V10, Note 5_gold_	V40, V10, Note 9, Note 5_gold_, Note 5_white_	−2~15	17
Inha Hi-Tech Center	V10, V40	V10, V40, Note 9, Note 5_gold_, Note 5_white_	−1~15	1
Wellcounty 4 Apartment	Note 9	Note 9	−2~30	1
Woomin Villa	V10	V40, V10, Note 5_gold_, Note 5_white_	−1~4	1

**Table 14:** Accuracy and sustainability of FloorPair.

Name of Building	MAE (Mean Absolute Error)/The Number of Tests				
	Experiment 1	Experiment 2	Experiment 3	Experiment 4	Experiment 5
Lotte World Tower	0/10	0 / 10	-	-	-
POSCO Tower-Songdo	0/34	0/34	0/34	-	-
COEX	0/12	0/12	0/12	0/12	0/12
HYUNDAI Department Store COEX Branch	0/30	0/30	0/30	0/30	0/30
Gangnam Station	0/6	0/6	0/6	0/6	0/6
Indeogwon Station	0/6	0/6	0/6	0/6	0/6
I-first Tower	0/42	0/42	0/42	0/42	0/42
Woojung Town	0/20	0/20	0/20	0/20	0/20
Star Tower	0/34	0/34	0/34	0/34	0/34
Inha Hi-Tech Center	0/32	0/32	-	-	-
Wellcounty 4 Apartment	0/64	0/64	-	-	-
Woomin Villa	0/10	0/10	0/10	0/10	-
